# Do clinical guidelines reduce clinician dependent costs?

**DOI:** 10.1186/1478-4505-9-24

**Published:** 2011-06-16

**Authors:** George Kosimbei, Kara Hanson, Mike English

**Affiliations:** 1 Child and Newborn Health Group, KEMRI/Wellcome Trust and School of Economics, Kenyattta University, P.O. box 43844, 00100 GPO, Nairobi, Kenya; 2 London School of Hygiene and Tropical Medicine, Keppel Street, London WC1E 7HT, London, UK; 3 Child and Newborn Health Group, KEMRI/welcome Trust and Department of Pediatrics, University of Oxford, P.O. Box 43640, 00100 GPO, Nairobi, Kenya

## Abstract

Clinician dependent costs are the costs of care that are under the discretion of the healthcare provider. These costs include the costs of drugs, tests and investigations, and discretionary outpatient visits and impatient stays. The purpose of this review was to summarize recent evidence, relevant to both developed and developing countries on whether evidence based clinical guidelines can change hospitals variable costs which are clinician dependent, and the degree of financial savings achieved at hospital level. Potential studies for inclusion were identified using structured searches of Econlit, J-Stor, and Pubmed databases. Two reviewers independently evaluated retrieved studies for inclusion. The methodological quality of the selected articles was assessed using the Oxford Centre for Evidence- Based Medicine (CEBM) levels of evidence. The results suggest that 10 of the 11 interventions were successful reducing financial costs. Most of the interventions, either in modeling studies or real interventions generate significant financial saving, although the former reported higher savings because the studies assumed 100 percent compliance.

## Introduction

Globally, health care expenditures have increased tremendously in the last decade raising concerns over their sustainability[1-4]and value for money. This increased investment is being made by both households and governments and represents a potential pool of resources that could be used elsewhere in the economy[2,4]. Such an increase in expenditure if resulting in improved health status would appear to be justified. However, there are concerns that extra spending on health is not yielding the anticipated health returns[1,5,6]. Therefore, there is an increasing interest in maximizing the efficiency of health care spending while at the same time increasing the effectiveness of service provision. One commonly used approach to improving the effectiveness of health care is to develop guidelines for the health care workers based on the best available evidence of what works[7-9]. Often such guidelines aim to improve outcomes through use of optimal treatment approaches and to reduce or limit costs by therapies or procedures. The degree to which guidelines work to change practices depends usually on the extent to which health workers change their behaviour in response to the guidelines as suggested in the Grimshaw 2004 review[7].

The cost consequence of such interventions has received less attention in the literature. The degree to which costs can be expected to change in response to clinical guideline adoption will depend on both the responsiveness of health worker behaviour, and on the share of patient costs that are attributable to decisions made by health workers. We have coined the term "clinician-dependent costs"****to describe the costs of care that are under the discretion of the healthcare provider. These costs include the costs of drugs, tests and investigations, and discretionary outpatient visits and inpatient stays. Changes in CDCs may arise from interventions such as clinical guidelines that aim to alter health workers use of available resources. We are therefore interested in examining the evidence that supports or refutes the proposition that CDCs can be altered by interventions aimed at changing health worker practices.

## Methods

This review aimed to address the question of whether and to what degree could interventions change costs that are clinician dependent. The particular focus was on studies reporting interventions designed to act through clinicians' behaviour change. We did not include studies describing the modeled difference in costs of care after assuming one drug or combination of drugs were substituted by an alternative drug or combination of drugs of equal or varying efficacy. Similarly we did not include studies that reported the cost-difference (or cost effectiveness difference) between alternative drug regimens whose efficacy was being compared in randomized controlled trials. Rather the focus was on interventions that aimed at influencing the clinician to follow a recommended process or procedure expected to influence CDCs. In this case the effect on costs was influenced by both the actual cost of the resources used and the degree to which the clinician opts to follow the advised procedures. Specifically this paper examines the degree of financial savings attributed to the interventions identified together with the resultant magnitude expressed as the proportionate reduction in total or consumable costs.

Potential studies for inclusion were identified by direct searches of the online databases such as; PubMed, J-Stor and Econlit and the grey literature to ensure a comprehensive literature review. The grey literature was sought at several libraries including those of the Kenya Medical Research Institute, Kenya Institute for Public Policy Research and Analysis, Institute of Policy Analysis and Research and the African Population and Health Research Center, Searches were also conducted through the websites of: The World bank, Department for International Development, United States Agency for International Development, Policy Project- Kenya, Health Policy Initiative and German Agency for Technical Co-operation (GTZ). Literature retrieved for the period 1995 to 2010 using the following combination of search terms:

■ Clinical guidelines and cost savings

■ Clinical prescribing behaviour and costs

■ Costs, decision making and prescribing variation

■ Prescription behaviour and costs

■ Prescribing behaviour and financial/costs savings

The titles and abstracts of the retrieved articles were read by two independent reviewers and those that met the pre-inclusion criteria were selected (Additional file 1, Tables S1 and S2). The methodological quality of the selected articles describing interventions was assessed using the Oxford Centre for Evidence Based Medicine (CEBM) levels of evidence, which rank the validity of evidence in a hierarchy of levels, with systematic reviews as level 1 (strong evidence) and expert opinions as level 5 (weak evidence)[10] (Additional file 1, Table S3).

## Results

The search terms used provided the following hits in PubMed, J-Stor and Econlit. In total 874 titles were screened, with 67 papers being reviewed. On reviewing these articles only 16 were considered for this structured review because they were truly related to our study question. The papers are described in Tables 1, 2 and 3 and in Additional file 1; Tables S1 and S2. Four studies report on models reflecting the anticipated effects of an intervention only ("modeling studies"). Eleven report the results of true intervention studies ("intervention studies") we present and discuss all of these studies.

**Table 1:** Modeling studies

Reference/Design	Country/setting	Inclusion criteria/intervention	Degree of financial saving	LOE
**Zurovac et-al. (2006); Modeling study/Secondary **data[12]	Kenya	Malaria microscopy is a potential solution to increase diagnostic specificity and overcome the problem of malaria over diagnosis	Costs of antimalarials decreased by 54% and those antibiotics increased by 5%	5

**Boulanger et-al. (1999); Cross-sectional survey**[13]	Kenya	IMCI guidelines would change drug prescribing behaviour	Compliance with guidelines could have reduced the cost of treatment by 42%	5

**Kolstad et-al. (1998); Cross-sectional survey**[14]	Uganda	IMCI guidelines would change drug prescribing behaviour	Use of IMCI could reduced costs of medication to US$0.17 per child from US$0.82 (A reduction of 80%)	5

**Khan et-al. (2002); Cross-sectional survey**[15]	Bangladesh	IMCI guidelines would change drug prescribing behaviour	Adoption of IMCI would reduce costs by 34%	5

LOE: Oxford Centre for evidence - Based Medicine level of evidence (May 2001)[10]

**Table 2:** Intervention studies (Change in cost due to change in actual guidelines)

Reference/Design	Country/setting	Inclusion criteria/intervention	Degree of financial saving	LOE
**Newton and Young (2006); Before and after study**[16]	USA	Inpatient diabetes management programme	Reduction in length of stay for patients with diabetes. Has resulted in savings of more than $2 million for the year and has yielded a 467% return on investment	2b

**McMullin et-al. (2005); Intervention and control**[23]	USA	Use of commercially available electronic prescribing system with integrated clinical decision support and evidence based message capability	The proportion of prescriptions for high cost drugs that were the target of this intervention to prescribers was a relative 17.5% lower among the intervention group compared with the control group	2b

**Wong et-at. (2000); Intervention and control**[22]	Hamilton Ontario	Multidisciplinary clinical pathway for oxygen management	Total health system costs increased by 116%	2a

**Hanna et-al. (1999); Before and after study. Before data was retrospective**[18]	USA	Use of a clinical pathway for patients undergoing total laryngectomy	The average hospital variable cost decreased from £3992 to £3419 per case. This represents a 14.4 reduction in costs associated with pathway implementation	2b

**Roth et-al (2001); Before and after study**[20]	USA	Educational intervention to decrease use of selected expensive medications	Annual saving of £66000 representing 32% decrease in use of more costly anti- coagulant and a 20% increase in the use of the less costly anticoagulant	2b

**Watson et-al. (2001); Cluster randomized trial**[26]	Avon England	Evaluate the effectiveness of guidelines with or without one to one educational outreach visits by community pharmacists	Mean costs reduced by 6% in practices that received mailed guidelines and educational outreach	1b

LOE: Oxford centre for Evidence-Based Medicine level of evidence (May 2001)[10]

**Table 3:** Intervention studies (Change in costs due to moving from incorrect to correct management)

Reference/Design	Country/setting	Inclusion criteria/intervention	Degree of financial saving	LOE
**Adam et-al. (2005); Comparative study**[11]	Tanzania	Districts with Integrated Management of Childhood Illness (IMCI) against those without	Cost per child in IMCI district was 44% lower than in district without IMCI. Although drug costs were higher by 61% in IMCI districts	2b

**Hogg et-al. (2005); Randomized control trial**[24]	Ontario Canada	Multifaceted intervention to improve preventive care delivered by nurses	Savings from a reduction in inappropriate testing were 35% of total health system costs	1b

**Ripouteau et-al. (2000); A controlled prospective before and after study**[17]	France	Multifaceted intervention to promote early switch from acetaminophen for prospective pain intravenous to oral	Mean cost per patient for analgesia decreased from £14 to £6 after the intervention to a 57% decrease	2a

**Boyter et-al. (1995); Before and after study**[19]	Britain	New antibiotic protocols, involving Amoxicillin as a first line agent	Mean consumable cost per patient reduced significantly from £14-09 to £10.20 this translates to a 28% reduction	1b

**Palmer et-al. (2000); Cluster randomized Control trial**[25]	Canada	Use of a critical pathway designed to manage community-acquired pneumonia more efficiently than conventional therapy	The pathway produced cost savings of 16%, 24% and 24% for the three perspectives respectively	1b

LOE: Oxford centre for Evidence-Based Medicine level of evidence (May 2001)[10]

### Study context and design

This review included a total of 15 studies, 4 being modeling studies and 11 being intervention studies. Intervention studies included examined changes in CDCs after implementation of an intervention aimed at changing clinician prescribing behaviour and are the main focus of this report. Study setting varied with 10 of 11 intervention studies conducted in high income countries and only one in low income setting[11]. All of the modeling studies (4) were conducted in low income countries[12-15] (see Table 1).

The study designs of intervention studies included comparative before and after studies[16-21], randomized trials[11,22] a comparative study (with a non-random allocation of study units)[23,24], and cluster randomized trials[25,26]. Of these the randomized trials were expected to provide the highest quality data with which to answer the question posed. The modeling studies are based on secondary data and all compared to theoretical scenarios with or without a complete change of clinical practice[12-15].

The level of evidence for economics and decision analysis as provided by the Oxford Centre for Evidence Based Medicine is provided in Additional file 1, Table S3. These criteria were used to evaluate the 11 intervention studies that were included in this structured review. A concise summary for both modeling and intervention studies containing information on study design, setting, intervention, the degree of financial saving attributed to the intervention and level of evidence are provided in Tables 1, 2 and 3 immediately after the main text.

### Interventions

The interventions reported by studies included in this structured review varied to a high degree although all interventions aimed at, or modeled changes in clinician behaviour through the use of clinical practice guidelines.

Interventions included: introduction of integrated management of childhood illness (IMCI)[11] clinical guidelines, a multifaceted intervention to improve preventive care delivered by nurses[24], a multidisciplinary clinical pathway for oxygen management[22], a multifaceted intervention to promote early switching from intravenous to oral acetaminophen for post-operative pain[17], introduction of new antibiotic protocols is the treatment of pneumonia[19], an educational intervention to decrease use of selected expensive medications[20], use of clinical pathway designed to manage community acquired pneumonia more efficiently than with conventional therapy[25], use of commercially available prescribing system with integrated clinical decision support and evidence based message capability[23], clinical pathways for patients undergoing total laryngectomy[18], inpatient diabetes management program[16] and evaluation of educational outreach visits by community pharmacists[26] on the effectiveness of guidelines. These interventions aimed at changing clinician behaviour either through training, prompts, feedback, supervision or a combination of this process.

For example, the IMCI intervention study in Tanzania involved an eleven day training period of which 30% of the time was spent on clinical practice with one follow up visit by an IMCI supervisor that occurred one month after training. This intervention changed prescribing behaviour which resulted in lower costs per child for the total resources used in treatment but increased drug costs[11]. In another study of a multifaceted intervention to improve preventive care delivered by nurses, the intervention was in the form of training[24], which covered an orientation session, medical office computer systems, medical practice management, prevention in primary care, evidence based medicine, and facilitator and audit skills development. The intervention was more expensive but more effective than any other attempts to modify primary care practice, since this intervention was targeted at changing the entire practice and not just physician behavior for a number of preventive measures and as a result more time was spent on site and more visits were required[24]. The study further suggests that the results of this study suggests that the results of this study can be considered an underestimate of the true potential costs savings given that not all the costs associated with inappropriate and appropriate preventive care were considered. Moreover, this study was an efficacy trial and as a consequence there is potential for reducing the cost of the intervention through efficiency improvements such as increasing the number of practices per facilitator as well as savings in administration and training through economies of scale. Another example comprises an intervention included in the study on multidisciplinary clinical pathway for oxygen management[22] which involved teaching sessions and individualized audit and feedback about oxygen ordering and monitoring. This intervention changed prescribing and monitoring practices but resulted in greater resource use, arguably improving the quality of care, with individual audit and feedback provided by the research nurse being most instrumental in achieving change[22]. The intervention on promoting early switch from intravenous to oral acetaminophen for postoperative pain included a local consensus process, short educational presentation, posters displayed in all nurses' offices, and feedback of practices six months after implementation of guidelines. This intervention leads to a reduction in irrational prescriptions. The final example of an intervention involved new antibiotic protocols which recommended the use of amoxicillin or erythromycin as a first line therapy for exacerbations of chronic obstructive airways disease (COAD). The protocols were effective because they resulted in significant reduction in costs, attributed to a shorter duration of therapy.

The studies based on modeling behavior change covered topics including: adherence to a practice guideline around malaria microscopy to overcome malaria over diagnosis[12] and appropriate implementation of a set of clinical practice guidelines in the form of the integrated management of childhood illness (IMCI) approach[13-15].

### Effectiveness of interventions and Compliance with guidelines

The success of any intervention aimed at altering clinician prescribing depends on compliance which translates to the effectiveness in changing health worker behavior. In a systematic review on effectiveness and efficiency of guideline dissemination and implementation strategies, clinical guidelines were found to result in observed improvements in care and absolute improvements in performance[7]. However, although the review reported significant effects of interventions the standardized mean differences in practice from intervention to control ranged from 0.9 to 6. This represents the magnitude of the change attributed to the intervention, including information on statistical significance. A summary of interventions and standardized mean differences is provided in Table 4 immediately after the main text and the information is obtained from Grimshaw (2004)[7]. In Table 4, standardized mean differences are not provided in some of the columns because there was insufficient data to facilitate its calculation.

**Table 4:** Effects of clinical guidelines as provided by standardized mean differences

Intervention	Source	Standardized mean difference (intervention and control)	Statistically significant
**Educational materials**	Randomized controlled trial	**+ 0.25**	Not Significant
	
	Cluster allocated controlled clinical trial	**None**	Not applicable
	
	Controlled before and after study	**+0.53**	Significant

**Educational Meetings**	Randomized controlled trial	**None**	Not applicable
	
	Cluster allocated controlled clinical trial	**None**	Not applicable
	
	Controlled before and after study	**None**	Not applicable

**Audits and feedbacks**	Randomized controlled trial	**None**	Not applicable
	
	Cluster allocated controlled clinical trial	**+0.2**	CCT
	
	Controlled before and after study	**None**	CBA

**Reminders**	Randomized controlled trial	**-0.28**	Not Ascertained
	
	Cluster allocated controlled clinical trial	**None**	Not applicable
	
	Controlled before and after study	**+0.15**	Not Significant

**Patient directed interventions**	Randomized controlled trial	**-0.67**	Significant
	
	Cluster allocated controlled clinical trial	**+6.00**	Significant
	
	Controlled before and after study	**+9.00**	Significant

**Other organizational interventions**	Randomized controlled trial	**+0.31**	Significant
	
	Cluster allocated controlled clinical trial	**None**	Not applicable
	
	Controlled before and after study	**-0.21**	Significant

*RCT: Randomized controlled trial, CBA: Controlled before and after study, CCT: Cluster allocated controlled clinical trial*.

This review further evaluates the success of interventions that are aimed at reducing clinician dependent costs, and thus generate some financial savings to the health system. On this note, interventions as opposed to modeling studies will be considered because they did not assume a high degree of compliance and they were more pragmatic. However, 10 out of the 11 interventions included in this review brought out the desired effect (See Table 5: as suggested by the reported degree of financial savings ranging from 6% to 57%). Only one study did not report any savings, and further suggested that multidisciplinary clinical pathway for oxygen management actually increased the costs of care by utilizing more resources[27]. In addition, an important element that deserves comment is the cost quality trade-off.

**Table 5:** Degree of Financial savings

Interventions studies	Degree of financial saving. {NB this is overestimated for any other denominator other than total health systems costs}	Proportion of total drug costs or total health system costs
**IMCI guidelines (Tanzania)**	Costs of drugs were higher by **61% **compared to control districts. Cost of the intervention not included.	Total drug costs for managing outpatient illness in those aged <5 years.
	
	Health system costs per child were **44% **lower in intervention districts than in control districts. Cost of the intervention was included.	Total health system costs (including start up and post implementation costs) for managing outpatient illness in those aged <5 years.

**Multidisciplinary clinical pathway for oxygen administration**	Led to changes in oxygen prescribing behaviour but consumed more resources than standard management by **116%**. Cost of the intervention was included.	Total health system costs (including start up and post implementation costs).

**Multifaceted intervention to improve preventive care delivered by nurses**	Costs decreased by **34%**. Cost of the intervention was included.	Total health system costs (training, Supervision)

**Use of commercially available electronic prescribing system with integrated clinical decision support and evidence - based message capability**.	The proportion of prescriptions for high costs drugs that were the target of this intervention to prescribers was a relative **17.5% **lower among the intervention group compared with the control group.	Total drug costs

**Multifaceted intervention to promote early switch from intravenous to oral acetaminophen for prospective pain**	Costs decrease by **57%**. Cost of the intervention not included.	Total cost for acetaminophen analgesia costs

**Clinical pathways for patients undergoing total laryngectony**	Hospital variable costs decreased by **14.4%**	Total hospital costs (both fixed and variable costs)

**New antibiotics protocol involving amoxicillin as a first line agent**.	Costs decrease by **28%**. Cost of the intervention not included.	Total antibiotics costs

**Educational intervention to decrease use of selected expensive medications**	A **32% **decrease in use of the more costly anticoagulant and a **20% **increase in use of the less costly anticoagulant representing an estimated annual savings of **$66000**. Cost of the intervention not included.	Total anti-coagulant costs

**Use of clinical pathway designed to manage community acquired pneumonia more efficiently than conventional therapy**	Cost savings of **16%, 24% **and **24% **from the perspectives: healthcare, government and societal. Cost of the intervention not included.	Total consumable costs

**Guidelines with one to one educational outreach visits by community pharmacists**	Mean costs reduce by **6%**.	Total drug costs including guideline production and dissemination; together with provision of education outreach visits

**In patient diabetes management program**	The reduction in length of stay for patients with diabetes has resulted in savings of more than **$2 **million for the year and has yielded a **467% **return on investment for the hospital.	Total hospital costs

**Modeling studies**	**Degree of financial saving**	**Proportion of total drug costs or total health system costs**.

**Malaria microscopy (change in clinical practice)**	Costs of prescribed Artemether-Lumefantrine decreased by **54% **while that of antibiotics increased by **5%**. Therefore, the overall drug cost saving was **49%**. Cost of the intervention not included.	Total Outpatient drug costs

**IMCI guidelines (Kenya)**	Cost of treatment reduced by an average of 42%. Cost of the intervention not included.	Total drug costs for managing outpatient illness in those aged <5 years

**IMCI guidelines (Uganda)**	Cost of medication reduced by **80%**. Cost of the intervention not included.	Total drug costs for managing outpatient illness in those aged <5 years

**IMCI guidelines (Bangladesh)**	Cost of treatment reduced by **34%**. Cost of the intervention not included.	Total drug costs for managing outpatient illness in those aged <5 years.

### Effects on Costs

The effects of interventions on clinician dependent costs, together with the cost areas forming the denominators, are presented in Table 5 immediately after the main text. Several studies have reported the degree of financial savings arising from certain interventions aimed at improving clinicians' prescribing behavior[11-15,17,19,20,22,24-26]. The studies which modeled the effect of changing health worker behavior present degrees of financial savings on CDC's limited to drug costs ranging from 26 to 80 percent. On the other hand, 10 of 11 true interventions studies, whose denominations varied from total drug costs to total health systems costs, reported financial savings ranging from 6 to 57 percent. Some studies don't provide clear information on whether they included intervention costs or not making the analysis rather difficult as evident in Table 5. However, in the study on a multidisciplinary clinical pathway for oxygen management an increase in total health systems costs by 116 percent[22] was reported whereas in the study of IMCI in Tanzania although an overall health system cost saving was reported this was despite an increase in drug costs by 61 percent[11].

## Discussion

In this section, prominent issues that surface from the reviewed studies are discussed. The variation in the denominator used in the costing approaches has clear implications when interpreting the resultant degree of financial saving. For example, financial savings presented as a percent of total drug costs may suggest a high degree of success of the intervention. However, such savings may be much less significant as financial savings presented as a percent of total health systems costs. The latter would obviously be preferred because it encompasses the total health system consequences as opposed to dealing with only drug costs. Furthermore, total health systems costs will include the marginal cost of the intervention which has implications on the output. We were able to identify only three of 8 studies that included total health systems costs when reporting the net savings. The variation in cost denominator used in currently published work therefore makes it very hard to compare or contrast different interventions and, other than suggesting generally positive results in terms of financial savings, limits the ability of current work to inform policy more generally. In the particular case evaluating the introduction of IMCI guidelines the change in perspective of the analysis, from direct resource cost savings to total health system costs results in a completely different interpretation of the impact of the intervention.

There are several determinants of cost savings which are highlighted in the studies included in this review. Some of these determinants are: minimizing laboratory tests and procedures, increasing diagnostic specificity, generic prescribing, use of cheaper and effective drugs, and increasing local financial responsibility (with the example of joining fund holding practices or primary care groups in a developed country). However, several issues stood out with respect to changing clinician dependent costs.

### The "room" or threshold to reduce costs

There is a possibility that studies are only conducted and reported in areas where they already feel that costs are unreasonably high[11,17,19,20,24,25], hence making it more likely that their intervention will work. This bias serves to show that clinical guidelines will always reduce costs, whereas there are many other reasons why guidelines may be helpful in improving quality of care. It is imperative that reduction of costs of financial savings should not be the goal of an intervention, rather issues such as quality of care.

### The uptake of the clinical guidelines

Modeling studies assume a 100 percent success rate whereas the intervention studies have a success rate of less than 100 percent. This gives intervention studies greater appeal for use in policy making because they use real life scenarios. Furthermore, cost effectiveness studies of various interventions would be desired provided quality of care is not disregarded. In this review, one real intervention study evaluating the multidisciplinary clinical pathway for oxygen management led to an increase in health care costs, but improved the quality of care significantly[22]. Besides, there is possibility that studies are conducted for interventions that people think will generate cost savings leading to the distinct possibility of not only publication bias but research initiation bias.

### The cost quality trade-off

Minimization of costs through use of cheap or poor quality resources may compromise health care quality. An optimal balance is required based on cost effectiveness of the particular intervention. It is necessary to compare the costs of an intervention with the effectiveness in order to justify resource use[28].

## Conclusions

This review is aimed at establishing the degree of financial saving on clinician dependent costs brought about by clinical guidelines. However, there was relatively little specific literature on the cost consequences of approaches to improving clinical practice despite the broad literature exploring means to change clinician behavior and improve practice. The methodological quality of the selected articles was assessed using the Oxford Centre for Evidence- Based Medicine (CEBM) levels of evidence and in general the quality of studies (summarized in Tables 1,2,3,5 and Additional file 1, Tables S1 and S2) was reasonably high and included several randomized controlled intervention trials. Eleven of the twelve interventions were successful in that they generated some financial savings (Table 5) however there is a possibility of publication bias in this regard plus the possibility that cost analyses are only undertaken if it can reasonably be expected that costs will be reduced. Although studies that involved a purely modeling approach often suggested significant cost reductions, they based these estimates on unrealistic assumptions of compliance or health worker behavior change and are consequently assigned a low level of evidence. These studies are likely to overestimate potential savings (Table 3). This drawback could be addressed by conducting sensitivity analysis with different compliance rates and emphasizing that the results are based on modeling assumptions closest to observed degrees of practice change.

## Competing interests

The authors declare that they have no competing interests.

## Authors' contributions

GK was responsible for literature search, selection of specific literature and production of the manuscript. KH was responsible for reviewing the draft manuscript, together with providing valuable comments and contributions. ME assisted in the selection of specific literature and provided significant comments in the development of the manuscript together with the funds which facilitated the production of this manuscript. All the authors read and approved the final manuscript.

## Supplementary Material

Additional File 1 **Table S1**. Levels of evidence for economic and decision analysis as provided by the Oxford Centre for Evidence- Based Medicine **Table S2 **Summary of studies included in the systematic review (Real interventions) **Table S3 **Summary of studies included in the systematic review (Modeling studies). Click here for file
