# Supplementary material for: Do clinical guidelines reduce clinician dependent costs?
Source: Health Res Policy Syst. 2011 Jun 16;9:24. doi: 10.1186/1478-4505-9-24 (PMC3128844; doi:10.1186/1478-4505-9-24)
Supplement: Additional File 1 — Table S1. Levels of evidence for economic and decision analysis as provided by the Oxford Centre for Evidence- Based Medicine Table S2 Summary of studies included in the systematic review (Real interventions) Table S3 Summary of studies included in the systematic review (Modeling studies). [file 1478-4505-9-24-S1.DOC]

**Additional File 1**

**Table S1** Summary of Studies included in the systematic review (Real interventions)

| **Methodological aspects/Study** | **Context** | **Objective** | **Setting** | **Design** | **Methods** | **Results** |
| --- | --- | --- | --- | --- | --- | --- |
| **Ripotaeu et-al, (2000) Effect of multifaceted intervention promoting early switch from intravenous to oral acetaminophen for postoperative pain: controlled, prosperous, before and after study[17]** | Need to improve the efficiency of postoperative pain management by early switching from intravenous to oral acetaminophen | Evaluate the effects of local guidelines aimed at improving nurses and doctors behaviour | Orthopaedic surgery department (intervention) and all other surgical departments (control) of a university hospital | A controlled, prospective, before and after study evaluated its impact on appropriateness and costs | The number of propacetamol injections peer patient and calculated the percentage of inappropriate injections during four audits performed in the orthopedic department at one month before and at one, three and six months after the intervention was implemented. | The mean cost per patient for acetaminophen analgesia decreased from £14 before the intervention to £6 after the intervention. We projected the annual cost reductions to be £15100. We estimated the cost of the intervention to be £970. The cost of the intervention was recuperated within three weeks. |
| **Watson et-al, (2001) Guidelines and educational outreach visits from community pharmacists to improve prescribing in general practice: a randomized controlled trial[26]** | Drugs prescribed in general practice account for over 10% of overall public expenditure on healthcare amounting to £4.7 billion in 1998. | To evaluate the effectiveness of guidelines with or without one to one educational outreach visits by community pharmacists in improving general practice prescribing for non-steroidal anti-inflammatory drugs (NSAIDs) | Avon, England | Cluster randomized trial | Practices were randomized to three groups: control, mailed guidelines, plus educational outreach visits. Changes in prescribing were measured using outcomes derived from PACT data. The primary outcome measure was change in the volume of prescribing for ibuprofen, declofenac and naproxen as a percentage of total NSAID prescribing. Six secondary outcomes included other measures of prescribing quality and volume. A cost benefit analysis was performed. | Mailed guidelines would lead to annual savings of £27.01 per GP, and the educational outreach would save £16.62 per GP. |
| **McMullin et-al, (2005) Twelve-month drug cost savings related to use of an electronic prescribing system with integrated decision support in primary care[23]** | To develop a follow-up report of a computerized decision support system (CDSS) | (i) Determine if the 6 months savings on new prescriptions were sustained during 12 months of follow-up (ii) evaluate the impact of the computerized decision support system (CDSS) on all pharmacy claims and per member per month expenditures and (iii) evaluate the prescribing behaviours within 8 high cost therapeutic categories. | The CDSS evaluation was conducted at Affinity health System (Menasha, WI). | Intervention and control study | Two database queries were performed to identify additional pharmacy claims data for all network health plan patients who were cared for by the 38 primary care clinicians included in the original 6 months study. | The proportion of prescriptions for high cost drugs that were the target of this intervention to prescribers was a relative 17.5% lower among the intervention group compared with the control group. |
| **Newton and Young (2006) Financial implications of glycemic control: Results of an inpatient diabetes management program[16]** | Length of stay for diabetic patients can be reduced to generate cost savings | (i) To determine the financial implications associated with changes in clinical outcomes resulting from implementation of an inpatient diabetes management program and (ii) to describe the strategies involved in the formation of this program. | Pitt Country Memorial Hospital, Greenville, NC, USA. | Before and after study | The various factors that influence financial outcomes are examine and previous and current outcomes are compared. | The reduction in length of stay for patients with diabetes has resulted in savings of more than $2 million for the year and has yielded a 467% return on investment for the hospital. |
| **Boyter et-al, (1995) Evaluation of an antibiotic prescribing protocol for treatment of acute exacerbations of chronic obstructive airways disease in a hospital respiratory unit[19]** | Introduction of a prescribing protocol for ineffective exacerbations of chronic obstructive airways disease (COAD) | Evaluating the impact of prescribing guidelines on length of stay and costs. | Hospital respiratory unit in the UK. | Matched two groups | The costs of the antibiotics prescribed to the sample of patients were calculated based on the actual price paid for the drugs by the hospital pharmacy. Also, the total cost of all antibiotics prescribed in the respiratory unit during the study period was calculated by obtaining pharmacy supply records. Data for each drug were converted to defined daily doses and the cost per DDD was calculated. | The average cost per DDD of all antibiotics prescribed in the respiratory unit fell from £3.77 in 1990 to £1.71 in 1991 (average reduction 54.6% P<0.05). |
| **Roth et-al, (2001) A simple institutional educational intervention to decrease use of selected expensive medications[20]** | Changing economics of health care delivery system demands that its resources be allocated wisely | To determine whether a simple educational intervention can influence use of prescription medications at an institution | A large urban fee standing academic rehabilitation hospital | Cost effectiveness analysis of prescribing behavior before and after an educational intervention | The hospital’s pharmacy department provided simple written educational material about cost differences of various prescription medications to attending and resident physicians, nurse managers. Telephoned reminders were given when targeted medications were prescribed. | A 32% decrease in the use of the more costly anticoagulant and a 20% increase in the use of the less costly anticoagulant representing an estimated annual savings of nearly $66000. |
| **Hanna et-al, (1999) Development and implementation of a clinical pathway for patients undergoing total laryngectomy[18]** | Development and implementation of a clinical pathway for patients admitted for a total laryngectomy | First, is to develop and implement the clinical pathway and second, to evaluate the impact of this pathway on the cost and quality of care of this patient population. | University of Arkansas for Medical Sciences, Little Rock, USA | Before and after study | A total of 45 patients were included in the study. The clinical pathway was implemented for 15 patients, white the 30 patients were treated without the implementation of the guidelines. | The clinical guidelines affected all the cost outcome measures. Length of stay decreased by 2.4 days, decreased by 2.4 days, and the average hospital variable cost decreased from $3992 to $3419 per case. This represents a 14.4% reduction in cost associated with pathway implementation. |
| **Palmer et-al (2000) Economic assessment of the community-acquired pneumonia intervention trial employing levofloxacin[25]** | Treatment of community acquired pneumonia (CAP) is very expensive especially for the impatient cases | To assess the use of a critical pathway designed to manage community-acquired pneumonia more efficiently than its management with conventional therapy | 19 participating Canadian hospitals | Randomized cluster design and controlled clinician trial. | Canadian hospitals were randomized to implement the critical pathway (n=9) or conventional theraphy (n=10). The critical pathway included a clinical prediction rule to guide the admission decision, treatment with levofloxacin, and practice guidelines. Patient data on medical resource use, lost productivity, and quality of life were collected prospectively for ≤6 weeks after treatment. Costs were calculated from the government, healthcare system and societal perspectives, with imputation of missing outpatient costs and the cost of lost productivity when necessary. | The critical pathway produced cost savings from all 3 perspectives (government, healthcare system and societal perspective that ranged from $457 to $994 per patient. |
| **Wong et-al (2000) Development dissemination, implementation and evaluation of a clinical pathway for oxygen therapy[22]** | Oxygen is commonly administered to patients in hospital, but prescribing and monitoring of such therapy may be suboptimal. | To develop and disseminate, implement and evaluate a multidisciplinary clinical pathway for the administration of oxygen. |  |  | The authors developed a clinical pathway for the ordering, titration and discontinuation of oxygen, which was disseminated through teaching sessions, in service training sessions and information posters in medical clinical teaching unit. Implementation of the pathway was ensured by means of reminders and patient- centered audit and feedback to CTU nurses and house staff. The intervention took three months, followed by a one month wash out phase by a three then by a three month non intervention phase. | In the intervention phase costs were higher for monitoring oxygen saturation ($44.95/ patient v. $36.17/ patient, p=0.048) and for order transcription ($2.71/ patient v. $1.28/ patient, p<0.001); total costs, including those for personnel, were also higher in the intervention phase ($76.93/ patient v. $56.67/ patient, p=0.02) The cost of education about the oxygen pathway was $45.71/ patient. When the education cost was included, the total cost of oxygen therapy during the intervention phase was $122.64/ patient; which was significantly higher that the total cost of oxygen therapy during the non-intervention phase ($56.67/ patient) (p<0.001) |
| **Adam et-al, (2005) Does the Integrated management of Childhood Illness cost more than routine care? Results from the United republic of Tanzania[11]** | IMCI has not been widely adopted partly because it is assumed to be more expensive than routine care | To compare the cost of IMCI with that of routine care in the four districts with and without IMCI | Four districts: Morogor rural and Rufiji districts were the intervention districts and Kilombero and Ulanga were the comparisons districts. |  | Total districts costs of child care were estimated from the societal perspective as the sum of child health- care costs incurred in a district at the household level, primarily health-facility level and hospital level, Administrative and support costs incurred by national and district administrations. The incremental costs of IMCI are the difference in costs of child health- care between districts with and without IMCI. | The annual cost per child of caring for children less than five years old on districts with IMCI was US$11.19, 44% lower than the cost in the districts without IMCI (US$16.09). After accounting for confounding, the difference in cost would be 6% lower. |
| **Hogg et al, (2005) Cost savings associated with improving appropriate and reducing inappropriate prevalence care: cost- consequence analysis[24]** | The net cost savings of implementing clinical preventive care guidelines need to be understood. | Estimate the proportion of a facilitation intervention cost that is offset and the potential for savings by reducing inappropriate screening tests and increasing appropriate screening tests in 22 intervention primary care practices | Southern Ontario |  | Cost consequence analysis of one successful outreach facilitation intervention was done taking into account the estimated cost savings to the health system of reducing five inappropriate tests and increasing seven appropriate tests. | The savings from reduction in appropriate testing were $148,568 and from avoiding treatment costs as a result of appropriate testing were $455,464 for a total savings of $604,032. On a yearly basis the net cost saving to the government is $191,733 per year (2003$Can) equating to $3, 687 per physician or $63,911 per facilitator, an estimated return on intervention investment and delivery of appropriate preventive care of 40%. |

**Table S2 Summary of studies included in the systematic review (Modeling studies).**

| **Methodological aspects/study** | **Context** | **Objective** | **Setting** | **Design** | **Methods** | **Results** |
| --- | --- | --- | --- | --- | --- | --- |
| **Zurovac et al, (2006) The financial and clinical implications of adult malaria diagnosis using microscopy in Kenya[12]** | Potential benefits of malaria microscopy are not realized because of irrational clinical practices and the low accuracy of routine microscopy | Model financial and clinical implications of revised clinical practices and improved accuracy of microscopy among adult outpatients under the artemether-lumefantrine (AL) treatment policy for uncomplicated malaria in Kenya. | 17 health facilities in Kenya |  | The cost of AL, antibiotics and malaria microscopy and the expected number of malaria diagnosis errors were estimated per 1000 adult outpatients presenting at a facility with microscopy under three scenarios A,B and C. | The costs of AL, antibiotics and malaria microscopy decreased from $2154 under option A to $1254 under option B and $892 under option C. Of the cost savings from option C, 72% was from changes in clinical practices, while 28% was from improvements in the accuracy of microscopy. |
| **Boulanger et al, (1999) Treatment in Kenyan rural health facilities: projected drug costs using the WHO-UNICEF IMCI guidelines[13]** | Guidelines for IMCI in peripheral health facilities have been developed by WHO and UNICEF to improve the recognition and treatment of common causes of childhood death. | To evaluate the impact of IMCI guidelines on treatment costs | All health facilities in Bungoma and Vihiga districts in Kenya. |  | Compared the cost of drugs actually prescribed to a sample of 747 sick children aged 2-59 months in rural health facilities in western Kenya with the cost of drugs had children been managed using IMCI guidelines. | The average cost of drugs actually prescribed per child was US$0.44 (196 US$). Antibiotics were the most costly component, with phenoxymethylpencillin syrup accounting for 59% of the cost of all drugs prescribed. Of the 295 prescriptions for phenoxymethylpenicillin syrup, 223 (76%) were for treatment of colds of drugs that would have been prescribed had the same children been managed with the IMCI guidelines ranged from US$ 0.16 per patient (based on a formulary of larger dose tablets and a commercial cough preparation). Compliance with existing treatment guidelines for the management of acute respiratory infections would have halved the costs of drugs prescribed. |
| **Kolstad et al, (1998) Potential implications of the integrated management of childhood illness (IMCI) for hospital referral and pharmaceutical usage in western Uganda[14]** | Medical assistant’s use of IMCI algorithms | Examine medical assistants’ use of IMCIU guidelines and compare how treatment following IMCI guidelines would differ from that which was prescribed by the hospital medical officers. | Kaborale district hospital, Fort Portal, Uganda |  | Compared medical officers’ recommendations with those of the IMCI. | The IMCI guidelines would have dispensed drugs to 1133 children at a total cost of US$204.73 or US$0.17 per child. However, for the same children medical officers prescribed drugs costing US$1003.68 or US$0.82 per child, a difference of 390%. |
| **Khan et al, (2002) Adopting integrated management of childhood illness module at local level in Bangladesh: implications for recurrent costs[15]** | Policy makers need information on cost to decide whether a new approach of managing child health will be feasible from financial point of view. | Examine recurrent cost associated with adoption of the IMCI module at first level health facilities in Bangladesh. | Matlab, Bangladesh |  | Experiment site of ICDDR, B Centre for health and Population research, Matlab, Bangladesh. | Adoption of IMCI should save about US$7 million on drugs. Prosper implementation of IMCI will require employment of additional health workers that will cost about US$2.7 million. If the current level of healthcare use is assumed, introduction of IMCI in Bangladesh will save over $4 million. |

**Table S3 Levels of evidence for economic and decision analysis as provided by the Oxford Centre for Evidence-Based Medicine[10]**

| **Level** | **1a** | **1b** | **1c** | **2a** | **2b** | **2c** | **3a** | **3b** | **4** | **5** |
| --- | --- | --- | --- | --- | --- | --- | --- | --- | --- | --- |
| **Explanation** | SR (with homogeneity) of level 1 economic studies | Analysis based on clinically sensible costs or alternatives: systematic review(s) of the evidence; and including multi-way sensitivity analysis. | Absolute better-value or worse value analysis | SR (with homogeneity) of level >2 economic studies | Analysis based on clinically sensible costs or alternatives: limited review(s) of the evidence, or single studies; and including multi- way sensibility analysis. | Audit or outcomes research | SR (with homogeneity) of 3b and better studies. | Analysis based on limited alternatives or costs, poor quality estimates of data, but including sensibility analysis incorporating clinically sensible variations. | Analysis with no sensitivity analysis | Expert opinion without explicit critical appraisal, or based on economic theory or “first principles” |
